# Electrocardiographic markers of increased risk of sudden cardiac death in patients with COVID‐19 pneumonia

**DOI:** 10.1111/anec.12824

**Published:** 2021-01-19

**Authors:** Mohammed Alareedh, Hussein Nafakhi, Foaad Shaghee, Ahmed Nafakhi

**Affiliations:** ^1^ Internal Medicine Department, Medicine College University of Kufa Najaf Iraq; ^2^ Internal Medicine Department Faculty of Medicine Jabir Ibn Hayyan Medical University Kufa Iraq; ^3^ Research Unit Najaf Health Bureau Ministry of Health Najaf Iraq

**Keywords:** COVID‐19, ECG, markers, pneumonia, sudden death

## Abstract

**Background:**

Little is known about the role of ECG markers of increased risk of sudden cardiac death during the acute period of coronavirus disease 2019 ( COVID‐19) pneumonia.

**Objectives:**

To evaluate ECG markers of sudden cardiac death on admission, including the index of cardiac electrophysiological balance (iCEB) (QTc/QRS) and transmural dispersion of repolarization (TDR) (T from peak to end (Tp‐e) interval and Tp‐e/QTc), in patients with COVID‐19 pneumonia.

**Patients and methods:**

This cross‐sectional study included 63 patients with newly diagnosed COVID‐19 pneumonia who presented to the outpatient clinic or admitted to the respiratory care unit between August 20 and September 15, 2020. Forty‐six persons matched for sex and age were selected from data collected before COVID‐19 pandemic.

**Results:**

QRS and QTc showed a significant prolongation in patients with COVID‐19 pneumonia compared to the controls (87 vs. 78, *p* < .00, and 429 versus. 400, *p* < .00, respectively). After categorization of patients with COVID‐19 pneumonia into 3 groups according to the severity of pneumonia as mild‐moderate, severe, and critical groups, a decreased values of QRS were observed in the critical COVID‐19 pneumonia group compared to severe and mild‐moderate COVID‐19 pneumonia groups (*p* = .04) while increased values of QTc and iCEB(QTc/QRS) were noted in critical COVID‐19 pneumonia group compared to other 2 groups(*p* < .00).

**Conclusions:**

Patients with COVID‐19 pneumonia showed significant changes in repolarization and conduction parameters compared to controls. Patients with mild to severe COVID‐19 pneumonia may be at low risk for torsades de pointes development.

## 1 INTRODUCTION

Coronavirus disease 2019 (COVID‐19) is caused by severe acute respiratory syndrome coronavirus‐2 (SARS‐CoV‐2) which predominantly affects the lung and may progress to pneumonia, respiratory failure, and even death (Kwenandar et al., 2020; McCullough et al., 2020; Xiong et al., 2020).

Cardiac involvement and cardiovascular dysfunction are possible complications associated with COVID‐19 infection. Data from previous outbreaks and recent case reports have demonstrated the adverse effects of COVID‐19 on the heart, including myocarditis, heart failure, heart block, arrhythmias, and sudden cardiac death (Xiong et al., 2020; Yenerçağ et al., 2020).

A common cardiac manifestation in patients with COVID‐19 infection is disturbances of cardiac rhythm. In a cohort study of 137 hospitalized patients with COVID‐19 infection, 75 of enrolled patients complained of palpitation as a presenting symptom (Bacharova, 2019; Bertini et al., 2020) Furthermore, arrhythmias is one of the most known causes of death in critically ill patients. A higher rate of arrhythmic events was reported in patients with COVID‐19 infection particularly in those admitted to intensive care unit where the prevalence rate was double in comparison with non‐intensive care unit patients (16(44.4%) vs. 7(6.9%), *p* < .00; Wang et al., 2020) However, the specific type and potential underlying mechanisms are not defined and remained a challenging issue in clinical practice (Vlachakis et al., 2020).

The assessment of standard electrocardiography (ECG) changes and markers in COVID‐19 infection is still sparse and not fully defined (Angeli et al., 2020) Also, little is known about the role of ECG abnormalities and markers of increased risk of adverse cardiac events particularly during the acute period of COVID‐19 pneumonia (Angeli et al., 2020).

Recently, several novel electrocardiographic markers, including transmural dispersion of repolarization(TDR) of left ventricle, measured by T peak to end interval (Tp‐e) and Tp‐e/QTc, and index of cardiac electrophysiological balance (iCEB), measured by QTc/QRS, are proposed to be associated with increased risk of malignant arrhythmias and sudden cardiac death in several cardiovascular diseases (Yenerçağ et al., 2020; Al‐Mosawi et al., 2018; Nafakhi et al., 2018; Mandala & Di, 2017).

The present study aimed to assess the ECG markers of increased risk of malignant arrhythmias and sudden cardiac death on admission, including iCEB (QTc/QRS) and TDR (Tp‐e interval and Tp‐e/QTc), in patients with COVID‐19 pneumonia.

## 2 PATIENTS AND METHODS

This was observational cross‐sectional study included 63 patients with newly diagnosed COVID‐19 pneumonia who presented to the outpatient clinic or admitted to the respiratory care unit (RCU) at Al‐Amal (the Hope) specialized hospital for communicable diseases in Al‐Najaf governorate from August 20, 2020, to September 15, 2020. All patients were presented with features consistent with COVID‐19 pneumonia based on clinical symptoms and radiological findings (CXR or computerized tomography (CT) examination of the lung). The diagnosis of COVID‐19 was confirmed by polymerase chain reaction (PCR) nasopharyngeal swab. Patients with chronic kidney disease, anti‐arrhythmic medications, pacemaker implantation, and atrial fibrillation and uninterpretable ECG paper were not included in the present study.

The baseline clinical characteristics for each patient were recorded at the time of outpatient clinic visit or RCU admission, including age, sex, comorbidities, body mass index (BMI), and drug history. The control group consisted of 46 age‐ and sex‐matched persons who proved to have normal coronary arteries according to CT coronary angiography examination, having no lung disease, valvular or myocardial diseases. Controls were selected from our previous work collected before the COVID‐19 pandemic (Al‐Mosawi et al., 2018; Nafakhi et al., 2018). Approval of this study was provided by our medicine college board.

### 2.1 ECG examination

The 12‐lead ECGs were obtained for all patients at the time of outpatient clinic visit or within 24 hr of RCU admission with a paper speed of 25 mm/s and voltage of 10 mm/mV by using a standard ECG system (Marquette Electronics) while the patient was resting in the supine position. ECG readings were measured manually by two cardiologists blinded to the patient's status, using calipers and a magnifying glass. Any disagreement in ECG interpretations between cardiologists was resolved by consensus. QRS duration in milliseconds was measured from the initiation of the Q or R waves until the end of the R or S waves (Mandala & Di, 2017). Tp‐e interval in milliseconds was measured from the peak of the T wave to the end of the T wave in the precordial leads. The mean value of the measurements was used in the analysis. The QT interval in milliseconds was measured from the beginning of the QRS complex to the end of the T. Measured QT intervals were corrected by Bazett's formula (QT/(RR interval)1/2) and defined as corrected QT interval (QTc). The Tp‐e/QTc ratio and QTc/QRS were calculated from these measurements (Al‐Mosawi et al., 2018; Nafakhi et al., 2018).

### 2.2 Statistical analysis

Statistical analysis was performed using SPSS ver. 23.0 (SPSS Inc.,). Clinical data of the patients and ECG markers were expressed as mean ± standard deviation for continuous variables or as numbers with percentages for categorical data. Student's *t*‐test was used to assess the distribution of clinical patients' characteristics and ECG markers between the COVID‐19 pneumonia group and controls. Differences in clinical patients' characteristics and ECG makers among COVID‐19 pneumonia groups were evaluated using one‐way ANOVA test. *p*‐value of < .05 was chosen for statistical significance.

## 3 RESULTS

A total of 109 persons were enrolled in this study. Among these, 63 patients were diagnosed with COVID‐19 pneumonia and 46 persons as a healthy control group matched for age and sex to the COVID‐19 group. There were no significant differences between COVID‐19 group and control group in terms of BMI, hypertension, diabetes mellitus, and smoking status. The baseline clinical characteristics of COVID‐19 pneumonia group and control group are shown in Table 1.

**TABLE 1:** Patient's characteristics

	COVID−19 pneumonia *n* = 63	Control group *n* = 46	*p* value
Age(years), mean ± *SD*	49 ± 13	51 ± 11	.56
Male, %	28 (44%)	20 (43%)	.74
BMI, mean ± *SD*	30 ± 5	29.7 ± 4	.75
Hypertension,%	20 (31%)	20	.20
Diabetes mellitus,%	13 (21%)	6 (13%)	.32
Smoking,%	7 (11%)	6 (13%)	.75
ECG markers			
QRS duration (ms),mean ± *SD*	87 ± 12	78 ± 13	<.00
QTc interval(ms),mean ± *SD*	429 ± 36	400 ± 32	<.00
Tp‐e (ms),mean ± *SD*	69 ± 12	73 ± 11	.62
iCEB(QTc/QRS), mean ± *SD*	4.9 ± 0.8	5.2 ± 1	.08
TDR(Tp‐e/QTc), mean ± *SD*	0.15 ± 0.0	0.18 ± 0.0	<.00

Note

Abbreviations: BMI, body mass index; iCEB, index of cardiac electrophysiological balance; ms, milliseconds; *SD*, standard deviation; TDR, transmural dispersion of repolarization; Tp‐e, T from peak to end interval.

### 3.1 Assessment of ECG markers between COVID‐19 pneumonia group and control group

QRS duration and QTc interval showed a significant prolongation in COVID‐19 pneumonia group compared to control group (87 vs. 78, *p* < .00 and 429 vs. 400, *p* < .00 respectively). Decreased values of TDR (Tp‐e/QTc) were more prevalent among COVID‐19 pneumonia group compared to control group (0.15 vs. 0.18,*p* < .00). There were no significant differences in Tp‐e interval and iCEB(QTc/QRS) values between COVID‐19 pneumonia group and control group (*p* > .05) (Table 1).

### 3.2 Assessment of ECG markers among COVID‐19 pneumonia groups

According to the severity of COVID‐19 pneumonia, patients were assigned into 3 groups as mild‐moderate COVID‐19 pneumonia group consisted of 48 (76%) patients not requiring hospital admission and treated at home with lung injury < 50% on CT examination, severe COVID‐19 pneumonia group consisted of 10 (16%) patients with > 50% of lung damage on CT examination requiring admission to RCU, and critical COVID‐19 pneumonia group consisted of 5 (8%) patients with > 50% of lung damage on CT examination requiring mechanical ventilation within 72 hr from admission to RCU.

Patients with critical COVID‐19 pneumonia were significantly older and more likely to be male compared to the severe and mild‐moderate COVID‐19 pneumonia groups (68 vs. 54 vs. 49, *p* = .03 and 80% vs. 80% vs. 16, *p* < .00 respectively; Table 2).

**TABLE 2:** Distribution of ECG markers and patients' clinical characteristics among COVID‐19 pneumonia groups

	Mild‐moderate pneumonia (*n* = 48)	Severe pneumonia (*n* = 10)	Critical pneumonia (*n* = 5)	*p* value
Age(years),mean ± *SD*	49.7 ± 13	54 ± 12	68 ± 14	.03
BMI, mean ± *SD*	29.8 ± 5	31 ± 4	31 ± 5	.43
Male, %	16 (33%)	8 (80%)	4 (80%)	<.00
Hypertension, %	15 (31%)	3 (30%)	2 (40%)	.98
Diabetes mellitus, %	10 (21%)	2 (20%)	1 (20%)	.99
Smoking,%	5 (10%)	1 (10%)	1 (20%)	.81
ECG markers				
QRS duration(ms), mean ± *SD*	86 ± 11	96 ± 14	81 ± 6	.04
QTc interval(ms), mean ± *SD*	421 ± 33	447 ± 32	469 ± 44	.04
Tp‐e(ms), mean ± *SD*	70 ± 12	69 ± 14	62 ± 13	.42
iCEB(QTc/QRS), mean ± *SD*	4.9 ± 0.8	4.7 ± 0.6	5.8 ± 0.4	<.00
TDR(Tp‐e/QTc), mean ± *SD*	0.16 ± 0.0	0.15 ± 0.0	0.13 ± 0.0	.24

Note

Abbreviations: BMI, body mass index; iCEB, index of cardiac electrophysiological balance; ms, milliseconds; *SD*, standard deviation; TDR, transmural dispersion of repolarization; Tp‐e, T from peak to end interval.

A decreased value of QRS was observed in the critical COVID‐19 pneumonia group compared to the severe and mild‐moderate COVID‐19 pneumonia groups ( 81 vs. 96 vs. 86, *p* = .04). On the other hand, increased values of QTc and iCEB(QTc/QRS) were noted in the critical COVID‐19 pneumonia group compared to the severe and mild‐moderate COVID‐19 pneumonia groups (469 Vs. 447 vs. 421, *p* = .04 and 5.8 vs. 4.7 vs. 4.9, *p* < .00 respectively). No significant difference was observed in the distribution of Tp‐e interval and TDR values among COVID‐19 pneumonia groups (*p* > .5) (Table 2).

## 4 DISCUSSION

The finding of increased QRS duration and QTc interval in patients with COVID‐19 pneumonia is consistent with other studies (Antzelevitch, 2005; Bertini et al., 2020; Çınar et al., 1992).

A multi‐center analysis of 431 hospitalized COVID‐19 patients reported that QTc prolongation was the most frequent finding (38%) which can be attributed to critical illness and hypoxemia. Also, a significant prolongation in the QRS interval was found in 19% of enrolled patients, particularly among elderly patients, which can be attributed to right ventricular dysfunction in the context of respiratory failure even before starting drug therapy for COVID‐19 infection (Bertini et al., 2020). Other researchers also found that QTc were significantly increased in COVID‐19 patients compared to controls, while a review of 16 case reports related to COVID‐19 infection reported a high frequency of nonspecific intraventricular delay. (Öztürk et al., 2020; Liu et al., 2020).

In our study, the finding of decreased TDR observed in the COVID‐19 pneumonia group compared to controls is in disagreement with other studies.

Yenerçağ et al and M. Öztürk F et al have shown a significant increase in TDR(Tp‐e interval and Tp‐e/QTc) in newly diagnosed COVID‐19 patients compared to controls. (Çınar et al., 1992; Yenerçağ et al., 2020) However, lack of enrollment of patients with COVID‐19 pneumonia or lung involvement and controls before COVID‐19 infection were the main limitations of the above 2 studies.

It has been suggested that ECG markers may have a different predictive impact among different diseases (Mandala & Di, 2017). The prolongation of QTc interval may not increase the TDR and hence the risk of development of torsades de pointes in several clinical cases. Drugs that are associated with QTc prolongation with no increase in TDR have little or no potential to induce torsades de pointes but increased the risk of occurrence of classical ventricular tachycardia or ventricular fibrillation. As such, amiodarone induces preferential prolongation of the action potential duration associated with prolongation of QTc but TDR is reduced and torsades de pointes do not occur (Tse & Yan, 2017).

In the literature, iCEB(QTc/QRS) is a useful marker in predicting the risk of malignant ventricular arrhythmias and sudden cardiac death. In experimental studies, a variation (either increase or decrease) of iCEB values from baseline was reported to be associated with higher risk of ventricular arrhythmias (Bacharova, 2019). Robyns et al. reported iCEB potential diagnostic usefulness in differentiating the predisposition to torsades de pointes and non‐torsades de pointes, whereby the increase in the QTc/QRS ratio was associated with the predisposition to torsades de pointes, and its decrease was associated with the predisposition for non‐torsades de pointes‐mediated classical ventricular tachycardia or fibrillation (Robyns et al., 2016; Saleh et al., 2020). Hence, patients with mild‐moderate and severe COVID‐19 pneumonia groups who showed marked decreases in iCEB values, associated with relative prolongation of QRS and shortening of QTc compared to critical group, may be at lower risk for development of sudden cardiac death mediated by torsades de pointes type of ventricular tachycardia.

Taken together, these findings indicate that patients with COVID‐19 pneumonia may be at lower risk for development of torsades de pointes, as evident by decreasing TDR values compared to controls, but the risk of development of classical ventricular tachycardia/fibrillation is still present in mild‐moderate and severe COVID‐19 pneumonia groups, as evident by marked variation in iCEB (QTc/QRS) compared with controls and critical COVID‐19 pneumonia group.

Supporting these findings, a large reported cohort of patients with COVID‐19 that were treated with hydroxychloroquine with/without azithromycin reported that the risk of torsade de pointes was not increased in hospitalized COVID‐19 patients who showed a marked prolongation of QTc interval, particularly when combination therapy was used (Ankelson et al., 2020).

Furthermore, a cohort study of 90 patients hospitalized with clinical findings consistent with pneumonia reported marked QTc prolongation associated with one case of torsades de pointes and subsequently developed other ventricular arrhythmias. However, other confounding factors rather than QTc prolongation per se such as receiving hydroxychloroquine, myocarditis, and use of propofol, a drug that is known to induce torsades de pointes, might influence the occurrence of torsades de pointes in that case (Mercuro et al., 2020).

There are several limitations to the present study. The sample was relatively small, and we had incomplete data on medications for patients with critical COVID‐19 pneumonia, constituting 8% of the total COVID‐19 sample, prior to their admission to RCU, which could have a possible influence on ECG interpretation (e.g., transfer from other nearby centers to our specialized hospital in communicable disease with difficulty in obtaining previous medical records). These limitations did not allow us to conduct regression statistical analyses. We did not assess the heart by echocardiography or serum markers of myocardial damage to exclude myocardial disease. There was lack of data on serial ECG recordings or ECG data prior to the patient visit to outpatient clinic or admission to RCU. Controlling for all possible confounding factors, including the use of herbal or over the counter medicines or electrolytes disturbances before ECG recording, that might affect ECG changes was difficult. The marked increase of iCEB values and relative shortening of QRS duration in the critical COVID‐19 pneumonia group should be taken with caution because of a very small number of enrolled patients with critical status (5(8%)) and higher prevalence of male and elderly patients in the critical group than in other pneumonia groups, which may have led to a selection bias.

A large sample with a follow‐up study is required to confirm the results of the present study.

## 5 CONCLUSION

Patients with COVID‐19 pneumonia showed significant changes in repolarization and conduction parameters compared to controls. Patients with mild‐moderate and severe COVID‐19 pneumonia may be at low risk for the development of torsades de pointes, but the risk of classical ventricular tachycardia/fibrillation is still present in mild‐moderate and severe COVID‐19 pneumonia groups, as evident by decreased TDR and marked variation in iCEB values among COVID‐19 pneumonia groups.

## CONFLICT OF INTEREST

The authors declare that they have no conflict of interest.

## Data Availability

The data that support the findings of this study are available from the corresponding author upon reasonable request.
